# STAN and fetal deaths

**DOI:** 10.1111/aogs.14472

**Published:** 2022-10-17

**Authors:** Jostein Grytten, Ellen Blix, Irene Skau, Anne Eskild

**Affiliations:** ^1^ Department of Obstetrics and Gynecology Akershus University Hospital Lørenskog Norway; ^2^ Department of Community Dentistry University of Oslo Oslo Norway; ^3^ Faculty of Health Sciences OsloMet – Oslo Metropolitan University Oslo Norway; ^4^ Institute of Clinical Medicine University of Oslo Oslo Norway

Sir,

We thank Kessler et al. for their comments on our study.^1^

We carried out a population study with the aim to evaluate the impact of introduction of STAN in Norway on the following outcome measures: occurrence of fetal and neonatal deaths, Apgar scores <7, intrapartum cesarean sections and instrumental vaginal deliveries.^2^ We found no association between the introduction of STAN and any of the outcome measures, except for a small increase in the proportion of babies with Apgar scores <7.

In their Letter to the Editor, Kessler et al. conclude that “the data presented in the study do not support its key message”. The authors put forward several arguments to justify their conclusion. Below, we present their arguments, and give our response.

1. Kessler et al.^1^ argue that the relevant outcome measure is intrapartum stillbirth. We agree that this is a relevant outcome measure. Therefore, we have reanalyzed our data with intrapartum stillbirth as an outcome measure (Table 1). We present the results from three models (for details about model specifications see Appendix S1 in our original article by Blix et al.^2^). The results from the estimation of these models support our conclusion in our original article (Blix et al., tables 1 and 2).^2^
2. Kessler et al.^1^ question “whether the study has sufficient power to investigate hospital and time different effects of the ST analysis …”. We agree that the numbers are low. In particular, this is the case for intrapartum stillbirth, which is the authors' preferred outcome measure. In our data from the Medical Birth Registry, the total number of intrapartum stillbirths was 167 during 1989–2014. During 1989–1995 (before most of the birth units had introduced STAN), the mean number of intrapartum stillbirths was 7.4 per year.
  According to the authors, “the number of theoretically preventable neonatal deaths is substantially lower than its total count”.^1^ This is because “30%–40% of all neonatal deaths at term” cannot be prevented by STAN. Further “more than 60% of the deliveries had monitoring other than STAN”, and “cases delivered by cesarean section before the start of labor should be excluded”. Therefore, following the authors' line of reasoning, the total number of intrapartum stillbirths that potentially could have been prevented by using STAN during the delivery would be much less than 7.4 per year.
  It is difficult to estimate exactly how many intrapartum stillbirths could have been prevented. A reasonable estimate is that three to four intrapartum stillbirths could have been prevented yearly by STAN. This is a low number, in particular since there are about 55 000 deliveries each year in Norway. Also note that the regression coefficient is small (Table 1). During our study period, several other interventions were more effective in saving lives.^3^, ^4^, ^5^
3. Kessler et al.^1^ argue that “the decreasing intrapartum mortality in Norway, …, deserves an explanation”. In Figure S1, we show intrapartum mortality according to year. Graphs are shown for all birth units (top) and for the birth units in which STAN had been introduced during 1990–2014 (middle). Intrapartum mortality did not decrease. For each year, all the 95% confidence intervals overlapped. This may be one reason why we did not find an effect of STAN on our outcome measures.
  Note that the starting point for our graph in Figure S1 is 1989. This is the year from which data for elective cesarean sections were available in the Medical Birth Registry. The graph in the study by Kessler et al.^1^ starts in 1985.
4. Kessler et al.^1^ state that “we also miss abovementioned data for the units not using STAN”. We present intrapartum mortality for these units in Figure S1 (bottom). The mortality in units not using STAN is the same as in the units using STAN.
5. Kessler et al.^1^ suggest that the “paper provides little data on … confounding factors”. We disagree. In all our analyses we have included several variables that measure maternal risk factors for adverse pregnancy outcomes. These are well described in our original manuscript.^2^
6. Kessler et al.^1^ point out that “The gradual development of technology and guidelines … was not addressed”. This is not correct. In the original manuscript (Blix et al., table 4), we present results from a model in which we took into account that an effect of STAN may occur some years after its introduction.

**TABLE 1:** The effects of the use of STAN on intrapartum deaths during 1989–2014. The study population comprises singleton births, with gestational age ≥ 36 weeks, in 23 birth units that introduced the STAN technology. Elective cesarean sections are excluded

	Intrapartum deaths		
Regression model^a^	Model 1	Model 2	Model 3
Regression coefficient	−0.00006	−0.00007	−0.00006
Standard error	(0.00005)	(0.00056)	(0.00005)
*P*‐value	0.27	0.24	0.23
Model specification			
Maternal risk factors included	Yes	Yes	No
Linear trend (year)	Yes	Yes	Yes
Hospital fixed effects	Yes	Yes	Yes
Hospital fixed effects × linear trend	No	Yes	Yes
Number of intrapartum deaths^b^	122	122	129
Total^c^	923 110	923 110	960 045

^a^ Regression coefficients with standard errors clustered at the hospital level.

^b^ The numbers are lower in Models 1 and 2 because Model 3 does not adjust for maternal risk factors and thereby includes more women in the analysis.

^c^ Includes both liveborn and stillborn babies.

## Supporting information


Figure S1
