# Supplementary material for: STAN and fetal deaths
Source: Acta Obstet Gynecol Scand. 2022 Oct 17;101(12):1460–1. doi: 10.1111/aogs.14472 (PMC9812108; doi:10.1111/aogs.14472)
Supplement: Supplementary file 1 — Figure S1 [file AOGS-101-1460-s001.docx]

**FIGURE S1.** Intrapartum mortality according to year during 1989-2014. The study population comprises singleton births, with gestational age > 36 weeks. Elective cesarean sections are excluded.

All birth units included (n=46)


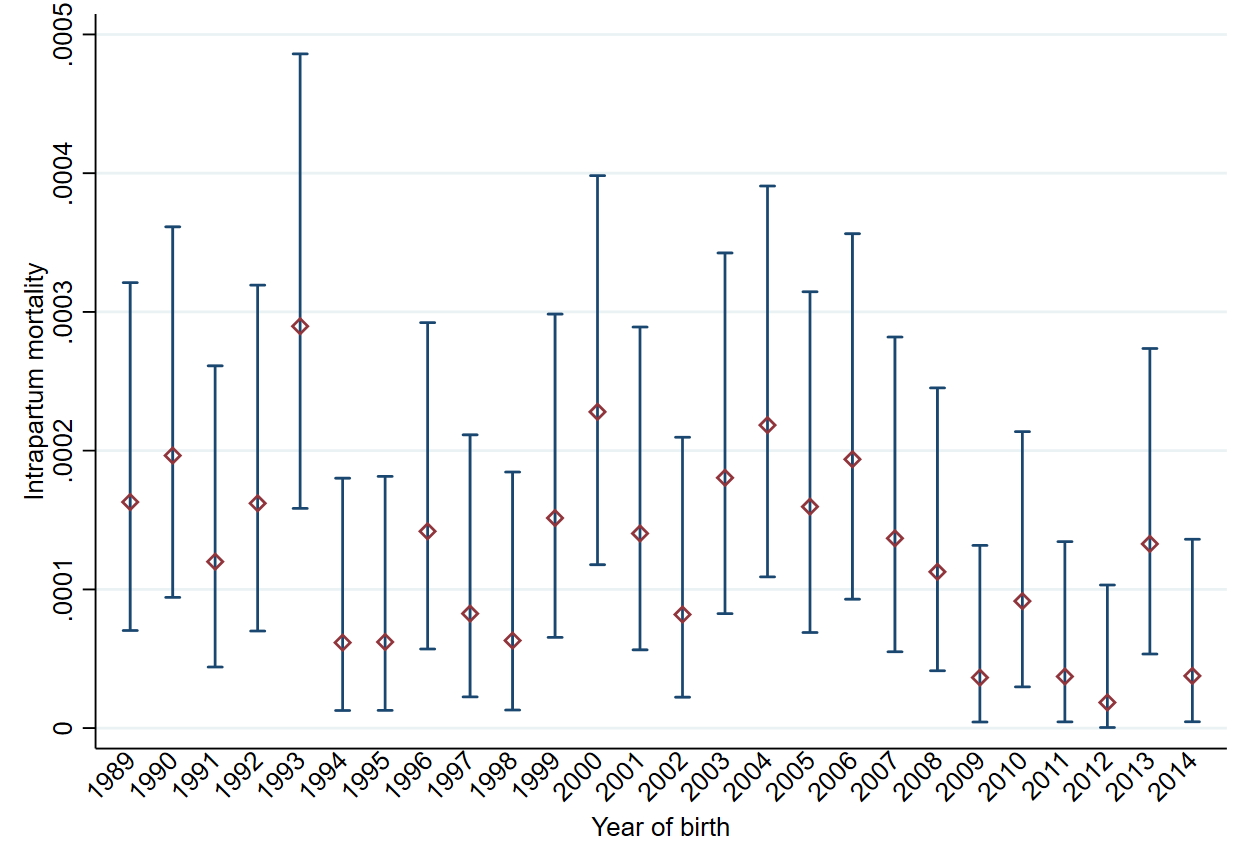


Birth units in which STAN had been introduced (n=23)


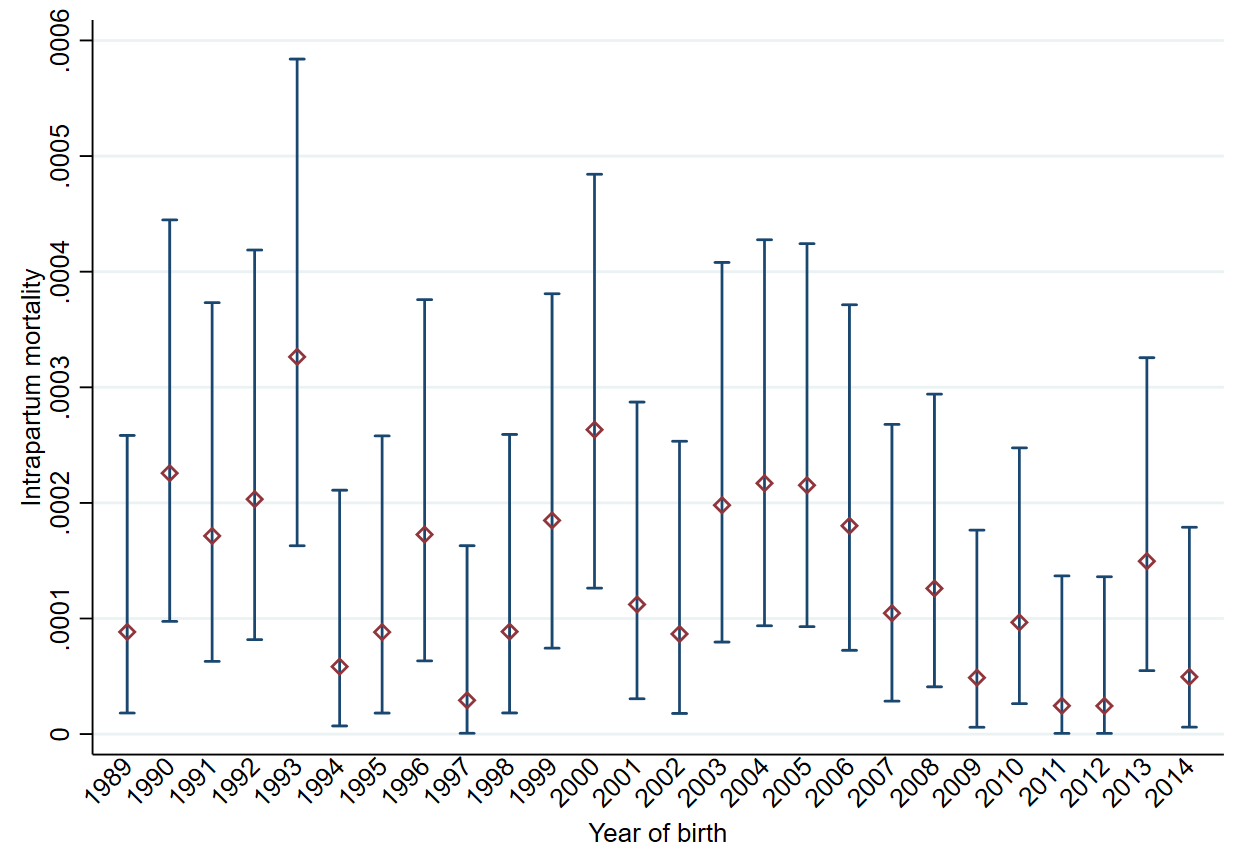


Birth units in which STAN had not been introduced (n=23)


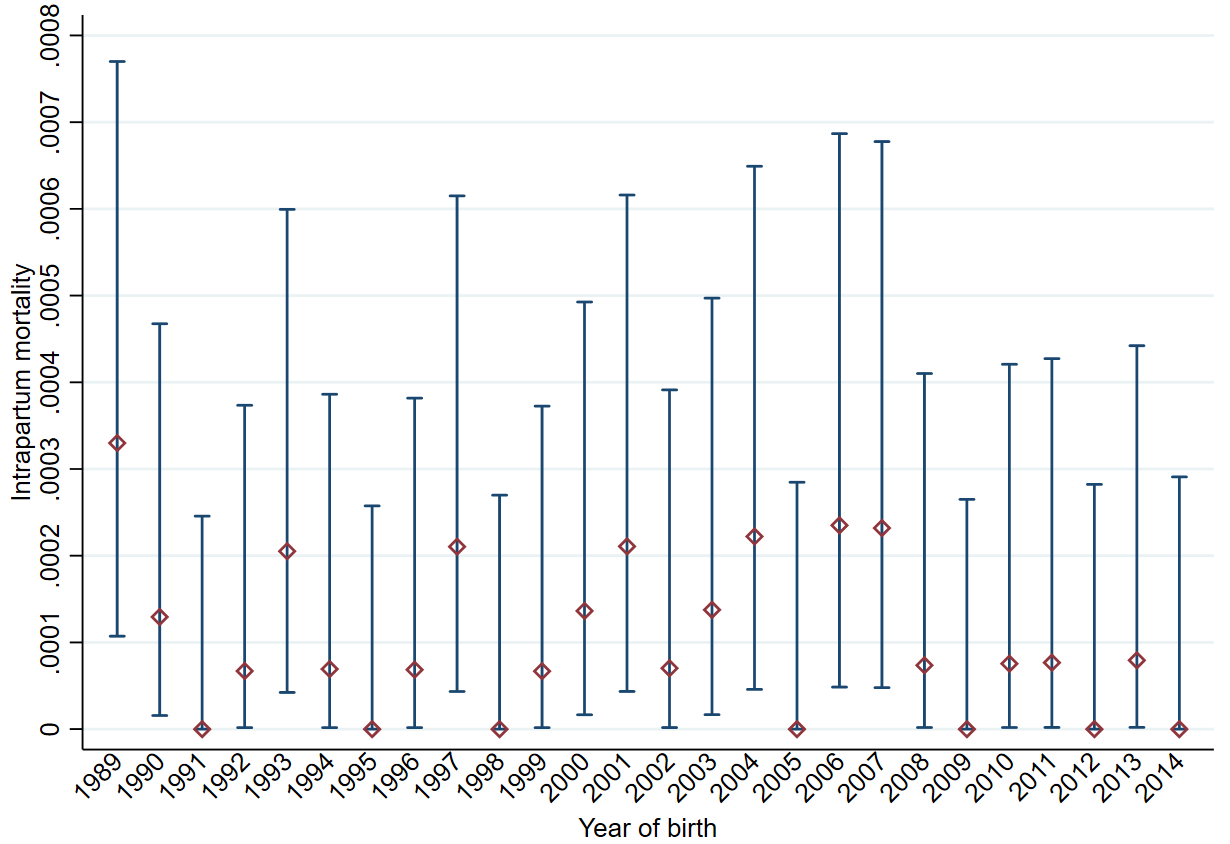


95% confidence intervals.
